# MAC-ErrorReads: machine learning-assisted classifier for filtering erroneous NGS reads

**DOI:** 10.1186/s12859-024-05681-1

**Published:** 2024-02-07

**Authors:** Amira Sami, Sara El-Metwally, M. Z. Rashad

**Affiliations:** 1 Department of Computer Science, Faculty of Computers and Information, Mansoura University, P.O. Box: 35516, Mansoura, Egypt; 2 Biomedical Informatics Department, Faculty of Computer Science and Engineering, New Mansoura University, Gamasa, 35712 Egypt

**Keywords:** Next-generation sequencing, Error filtration, Machine learning, Classification, Feature extraction

## Abstract

**Background:**

The rapid advancement of next-generation sequencing (NGS) machines in terms of speed and affordability has led to the generation of a massive amount of biological data at the expense of data quality as errors become more prevalent. This introduces the need to utilize different approaches to detect and filtrate errors, and data quality assurance is moved from the hardware space to the software preprocessing stages.

**Results:**

We introduce MAC-ErrorReads, a novel *M*achine learning-*A*ssisted *C*lassifier designed for filtering *Erro*neous NGS *Reads*. MAC-ErrorReads transforms the erroneous NGS read filtration process into a robust binary classification task, employing five supervised machine learning algorithms. These models are trained on features extracted through the computation of Term Frequency-Inverse Document Frequency (*TF_IDF*) values from various datasets such as *E. coli*, GAGE *S. aureus*, *H. Chr14*, *Arabidopsis thaliana Chr1* and *Metriaclima zebra*. Notably, Naive Bayes demonstrated robust performance across various datasets, displaying high accuracy, precision, recall, F1-score, MCC, and ROC values. The MAC-ErrorReads NB model accurately classified *S. aureus* reads, surpassing most error correction tools with a 38.69% alignment rate. For *H. Chr14*, tools like Lighter, Karect, CARE, Pollux, and MAC-ErrorReads showed rates above 99%. BFC and RECKONER exceeded 98%, while Fiona had 95.78%. For the *Arabidopsis thaliana Chr1*, Pollux, Karect, RECKONER, and MAC-ErrorReads demonstrated good alignment rates of 92.62%, 91.80%, 91.78%, and 90.87%, respectively. For the *Metriaclima zebra*, Pollux achieved a high alignment rate of 91.23%, despite having the lowest number of mapped reads. MAC-ErrorReads, Karect, and RECKONER demonstrated good alignment rates of 83.76%, 83.71%, and 83.67%, respectively, while also producing reasonable numbers of mapped reads to the reference genome.

**Conclusions:**

This study demonstrates that machine learning approaches for filtering NGS reads effectively identify and retain the most accurate reads, significantly enhancing assembly quality and genomic coverage. The integration of genomics and artificial intelligence through machine learning algorithms holds promise for enhancing NGS data quality, advancing downstream data analysis accuracy, and opening new opportunities in genetics, genomics, and personalized medicine research.

## Background

DNA sequencing data serves as a digital record of the nucleotide arrangement (A, C, G, and T) within a DNA molecule, playing a pivotal role in contemporary genomics research. DNA sequencing has evolved through generations. Sanger sequencing, representing the first generation, was labor-intensive and limited in volume. Next Generation Sequencing (NGS), the second generation (e.g., Illumina, Ion Torrent), enabled cost-effective high-throughput data generation. Third-generation technologies (e.g., Pacific Biosciences (PacBio), Oxford Nanopore) brought longer reads, increased speed, and cost reduction but with a trade-off of higher error rates [1–3].

NGS sequencing experiments generate a large volume of sequencing reads that require preprocessing, filtration, and mapping to a reference genome to uncover biological insights. Some reads fail to align uniquely and accurately to the reference according to different variations (substitutions, insertions, and deletions) and their origin from a repetitive region [2, 4, 5]. Variations impacting accurate mapping comprise true variations between reads and the reference (e.g., when sequencing similar genomes) and false variations, including errors from sequencing machines, contamination, and biases like PCR amplification during sequencing experiments [6, 7].

As sequencing machines become faster and more affordable, they generate extensive biological data, accompanied by errors that compromise data quality. This necessitates error detection and correction tools, shifting the responsibility from the hardware space to the software preprocessing stages. Most error detection and correction tools use two approaches: *k*-mer-based and alignment-based [8–11]. In the *k*-mer approach, reads are scanned by a window of size *k*, and *k*-mers are classified as strong or weak based on their frequency. While this approach is efficient, it introduces false positive corrections and requires intensive computational preprocessing tasks. Musket [12], Lighter [13], BFC [14], RECKONER [15], Blue [16], RACER [17], Pollux [18], and BLESS [19] are examples of error correction tools that follow this approach.

The second paradigm involves sequence alignment-based tools that organize the sequencing reads into an alignment matrix to maximize their similarity by aligning identical bases within the same column. Error correction tools based on sequence alignment consider the consensus of identical bases within each column, emphasizing high-frequency base coverage over low-frequency occurrences. Computing sequence alignment for high-throughput sequencing data is computationally intensive; some tools address this with heuristics and efficient data structures like hash tables, suffix trees/arrays, and de Bruijn graphs. Coral [20], ECHO [21], Fiona [22], Karect [23], Bcool [24], BrownieCorrector [25], and CARE [26] are examples of tools that follow the sequence alignment approach.

Machine learning is crucial in genomics to filter errors in NGS data, ensuring high-quality results. Machine learning algorithms play a pivotal role in identifying and removing sequencing errors, distinguishing true genetic variations from artifacts, and enhancing data accuracy. Automating data filtration with machine learning saves time and resources, allowing researchers to focus on the biological significance of the data rather than tedious error correction tasks [27, 28].

Many previously introduced error filtration approaches, based on machine learning techniques, rely on a complex computation of Multiple Sequence Alignment (MSA) and multiple *k*-mer hashing techniques as preprocessing stages [29, 30]. These approaches are designed to discern the accuracy of individual bases within sequencing reads by leveraging information gleaned from MSA, particularly when compared with similar sequences. The process of MSA involves the computation of nucleotide frequencies and the extraction of relevant information from adjacent columns. This information and factors such as quality scores and genomic coverage contribute to the formulation of features used in training machine learning models. Furthermore, other machine learning methods specialize in identifying the optimal *k*-mer size essential for independent error correction tools [31, 32] (see Table 1).

**Table 1:** Different machine learning approaches in sequencing data errors detection and correction

Machine learning models	Study goals	Pre-processing stage	Trained features	Ref
ANNs, NB, SVM, and 20 tree-based machine learning models	Classify each base in the metagenomics and polyploid samples as a true-variation base (signal) or an erroneous-variation one (noise)	Data clustering Multiple Sequence Alignment	The frequencies of the bases in each column in the resulted multiple sequence alignment matrix and in the cluster of the local similar sequences Windows with different distance thresholds to the intended classified base are constructed in order to consider the information available from its neighbors in the alignment matrix	[29]
RF	Utilized in CARE 2.0 error correction to classify each base in the low-quality multiple sequence alignment	*k*-mers Extraction and Hashing. Multiple Sequence Alignment	Local features:	[28]
			The relative frequency of the base being a candidate for correction	
			The quality-weighted relative frequency of the base being a candidate for correction	
			The average quality score of the base being a candidate for correction	
			The relative frequency of the consensus base	
			The quality-weighted relative frequency of the consensus base	
			The average quality score of the consensus base	
			The average quality score	
			The normalized coverage	
			The normalized average quality	
			Global Features:	
			The average quality-weighted relative frequency of the consensus base	
			The minimum quality-weighted relative frequency of the consensus base	
			The normalized maximum coverage	
			The normalized minimum coverage	
RNN	Choosing the best *k*-mer value for short reads error correction tools	Reads tokenization (Characters (i.e. bases) and Words (*k*-mers)) and transformation using one-hot encoding	The probability distribution of words (*k*-mers) and characters (bases) of the text (sequencing reads) considering their relative context	[30]
Transformer Network with self-attention	Choosing the best *k*-mer value for short and long reads error correction tools	Reads tokenization (Characters (i.e. bases) and Words (*k*-mers)) and transformation using sine and cosine positions encoding	The probability distribution of words (*k*-mers) and characters (bases) of the text (sequencing reads) considering their relative context	[31]

This paper introduces MAC-ErrorReads, a machine learning-assisted classifier designed to filter erroneous NGS reads. This innovative approach harnesses the power of machine learning to convert the process of erroneous NGS read filtration into a robust binary classification problem, where reads are classified as either ‘1’ for erroneous or ‘0’ for correct. MAC-ErrorReads was trained using five supervised machine learning algorithms: Naive Bayes (NB) [33], Support Vector Machine (SVM) [34], Random Forest (RF) [35], Logistic Regression (LR) [36, 37] and eXtreme Gradient Boosting (XGBoost) [38]. These algorithms were trained on a set of extracted features obtained through the computation of *TF_IDF* values for the set of identified *k*-mers from the sequencing data. The extracted features are computed without relying on expensive preprocessing stages such as MSA or multiple *k*-mers hashing techniques. To assess the efficacy and accuracy of these models, comprehensive testing and evaluation are conducted using simulated and real sequencing experiments. We employed stand-alone NGS alignment programs to evaluate the accuracy, precision, recall, F1-score, the Matthews correlation coefficient (MCC), and Receiver Operating Characteristic (ROC) metrics by comparing the predicted labels generated by MAC-ErrorReads with the true labels encoded in the alignment scores relative to the reference genome. Also, the correct classified reads were subjected to further downstream data analysis, involving genome mapping and assembly, to assess their respective mapping and assembly results. We expanded our analysis by assessing assembly results both with and without employing stand-alone error correction tools. Subsequently, we compared these results with those generated by the best reported machine learning model, both without error correction and after correcting the misclassified reads. Additionally, we benchmarked the correctly classified reads against those generated by various error correction tools, computing the total number of mapped reads and the overall alignment rate. The correct and false classified reads reported by the MAC-ErrorReads are subjected to redundancy analysis to identify the duplicated reads within both positive and negative sets.

MAC-ErrorReads makes the following contributions:

1. Most existing error filtration methods reliant on machine learning involve computationally intensive preprocessing stages, such MSA and multiple *k*-mer hashing techniques. Other machine learning methods specialize in identifying the optimal *k*-mer size essential for independent error correction tools. In contrast, MAC-ErrorReads eliminates the need for these costly preprocessing steps by computing *TF_IDF* values of identified *k*-mers from sequencing data, significantly reducing computational complexity.
2. MAC-ErrorReads simplifies the erroneous NGS read filtration into a robust binary classification problem. Instead of relying on complex preprocessing, it utilizes machine learning to categorize reads as '1' for erroneous or '0' for correct, ensuring efficiency and simplicity.
3. MAC-ErrorReads utilizes five supervised machine learning algorithms (NB, SVM, RF, LR, XGBoost) trained on features extracted from sequencing data using *TF_IDF* values of identified *k*-mers. The error filtration results are evaluated against various stand-alone error correction tools and one of the machine learning-based methods for error correction.
4. MAC-ErrorReads extends its analysis beyond error filtration to downstream processes such as genome mapping and assembly. It assesses the impact of correctly classified reads and correcting false ones using one of the stand-alone error correction tools on mapping and assembly results. This evaluation is conducted comprehensively through testing with both simulated and real sequencing experiments across different organisms.

## Methods

**Fig. 1:**
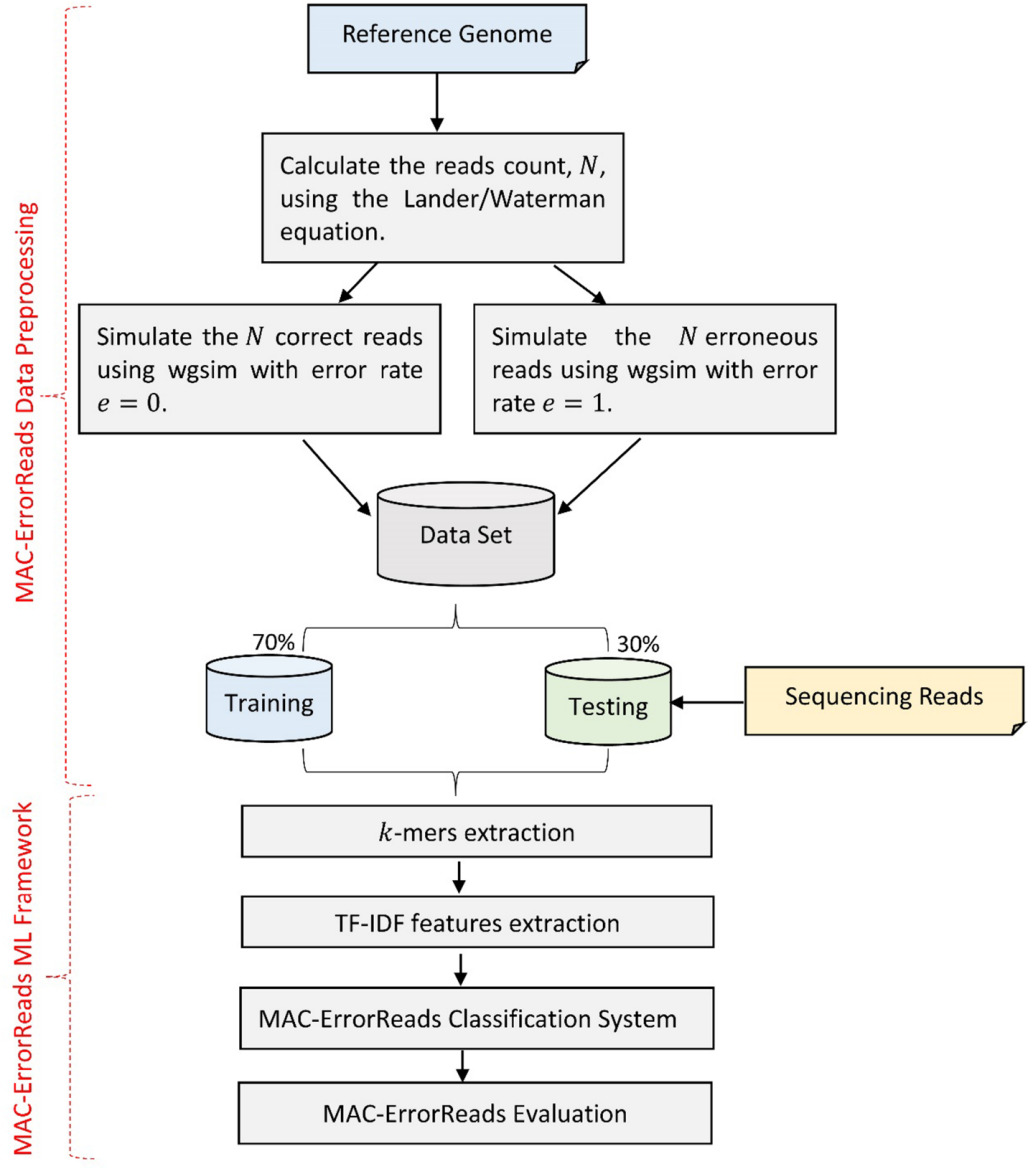
MAC-ErrorReads workflow for data preprocessing and classification

MAC-ErrorReads converts the process of filtering erroneous NGS reads into a machine learning classification problem. MAC-ErrorReads learns a mapping function F that transforms the input features space *X* extracted from each sequencing read into a binary label classification *Y* of this read as (1) for erroneous read and (0) for correct one. The MAC-ErrorReads workflow, depicted in Fig. 1, starts with preparing and preprocessing input data (reads) and assigning labels for training various machine learning models. The framework then extracts features by computing *TF_IDF* values for the *k*-mers derived from the labeled reads. These extracted features serve as inputs for training five distinct machine learning models. Subsequently, the models are tested and evaluated to determine their performance and accuracy, with detailed explanations of each stage provided in the following subsections.

### MAC-ErrorReads data preprocessing stage

The preprocessing stage started by preparing the labeled reads required for training machine learning models on a specific reference genome. Correct reads are labeled with 0, while the erroneous ones are labeled with 1. The wgsim [39] simulator, included within the SAMtools for whole genome simulation, generates the labeled reads for a reference genome with error rates equal *e* = 0 and *e* = 1 for correct and erroneous reads respectively. The total number of reads, *N*, that are required to cover a specific reference genome is computed in the proposed MAC-ErrorReads method as the following:

Suppose you have a reference genome with a length *G*, the genome is represented with a number of *N* randomly generated reads, and each read has a length of *L*. According to the Lander/Waterman equation for sequencing coverage, *C*, computation [40]:

$$C = \frac{LN}{G}$$

By knowing the genome length *G* and adjusting different parameters such as sequencing coverage *C* and read length *L*, we can expect the total number of *N* reads used to cover the genome randomly and subsequently used in the training process.

### MAC-ErrorReads *k*-mers and feature extraction stages

**Fig. 2:**
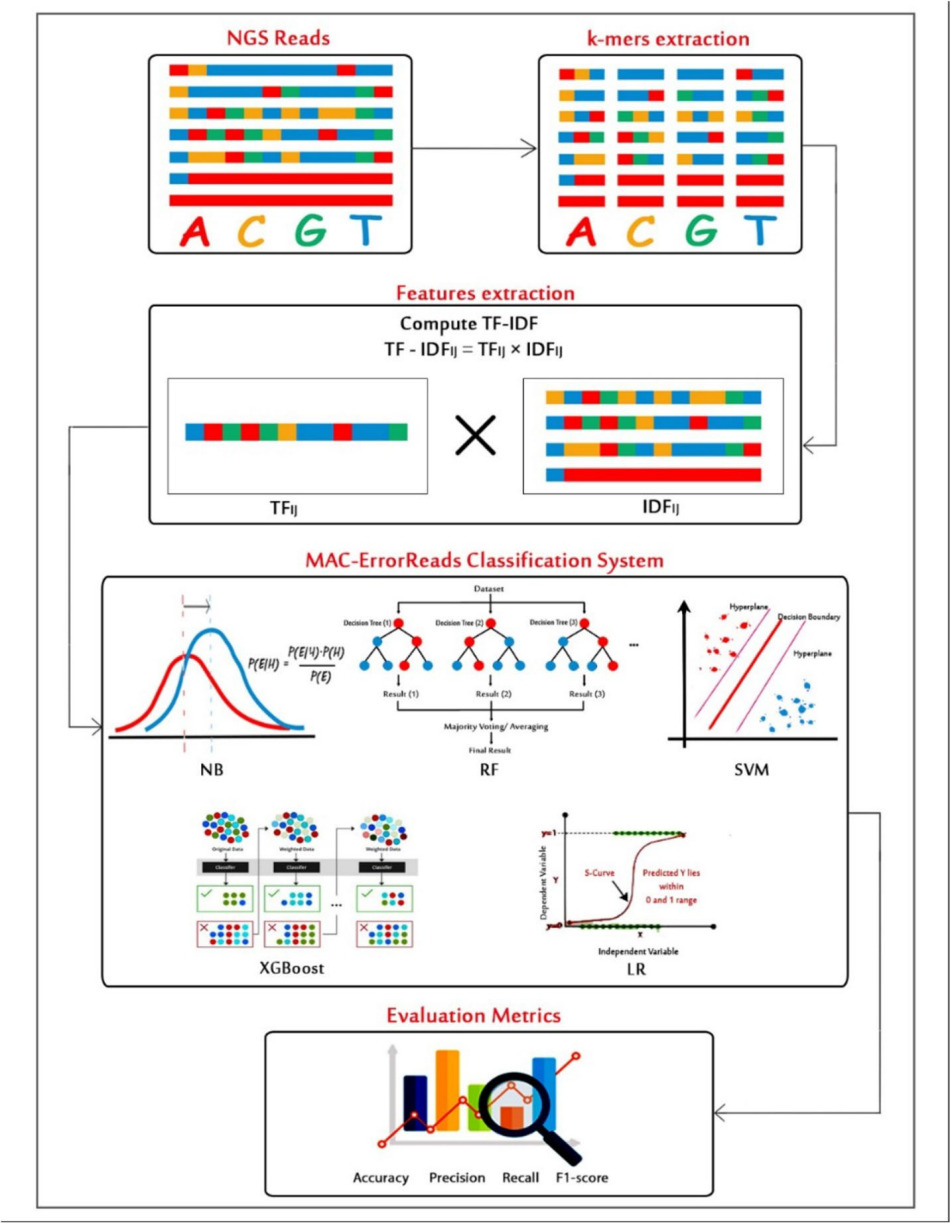
MAC-ErrorReads machine learning framework

After preparing the training dataset (labeled reads), the MAC-ErrorReads machine learning framework (see Fig. 2) begins by extracting *k*-mers from each labeled read. This is achieved by sliding a window of length *k* along the read sequence and capturing the *k*-mer at each position. The extracted *k*-mers are transformed into the set of *TF_IDF* values [41] that is used as training features for different machine learning algorithms. The *TF_IDF* is a numerical representation used in information retrieval and natural language processing to quantify the importance of a term in a document relative to a collection of documents. It is commonly used for text analysis and document ranking in search engines and information retrieval systems.

In this work, the sequencing reads are viewed as a set of documents, and the extracted *k*-mers are the terms that are quantified and analyzed. The *TF_IDF* score for a term (*k*-mer) in a document (sequencing read) is calculated based on two factors: Term Frequency (*TF*): This measures the frequency of a term (*k*-mer) within a document (sequencing read). It represents how often a particular *k*-mer appears in a sequencing read relative to the total number of *k*-mers in that sequencing read. The idea is that the more times a *k*-mer appears in a sequencing read, the more important it might be to that read sequence and consequently correct *k*-mer. The second factor is Inverse Document Frequency (*IDF*), which evaluates the significance of a term (*k*-mer) across a collection of documents (sequencing reads). It measures how rare or common a *k*-mer is in the entire sequencing dataset.

Suppose that a set of sequencing reads is *R*, with each read denoted as *r*_*i*_, where *i* ≤ *N*, and *N* is the total number of reads in the set *R*. Each read *r*_*i*_ has a set of extracted *k*-mers called *S*_*ij*_ where $j \le \left| {r_{i} } \right| - k + 1$. Each *k*-mer indicated as *s*_*ij*_ and its corresponding frequency in the read *r*_*i*_ is *f*_*ij*_.The *TF*_*ij*_ and *IDF*_*ij*_ scores are defined for each *k*-mer *s*_*ij*_ in each read *r*_*i*_, where *N*_*ij*_ is the number of reads *r*_*i*_ that have the *k*-mer *s*_*ij*_ in the whole dataset *R*.

$$\begin{aligned} TF_{ij} & = \frac{{f_{ij} }}{{\left| {S_{ij} } \right|}} \\ IDF_{ij} & = \log \left( {\frac{N}{{N_{ij} }}} \right) \\ \end{aligned}$$

The *TF_IDF*_*ij*_ score for a *k*-mer *s*_*ij*_ in a read *r*_*i*_ is calculated by multiplying the Term Frequency (*TF*_*ij*_) and the Inverse Document Frequency (*IDF*_*ij*_) of that *k*-mer:

$$TF\_IDF_{ij} = TF_{ij} \times IDF_{ij}$$

The result is a numerical value that reflects the importance of a *k*-mer in a particular read relative to the entire collection of sequencing reads. High *TF_IDF*_*ij*_ scores indicate that a *k*-mer is both frequent in the sequencing read and rare across the sequencing dataset, suggesting it is highly relevant to that read's content. Conversely, low *TF_IDF*_*ij*_ scores imply that the *k*-mer is common in the read or not particularly discriminative across the entire sequencing dataset. *TF_IDF*_*ij*_ is often used in text mining, document clustering, and information retrieval tasks to rank documents based on their relevance to a specific query or topic.

### MAC-ErrorReads classification system

The set of computed *TF_IDF* values represents a set of training features that feed into different machine algorithms for classifying each read as erroneous or error-free. The input sequences are classified into erroneous read (1) and correct read (0). The MAC-ErrorReads classification system utilizes five supervised machine learning algorithms: NB [33], SVM [34], RF [35], LR [36, 37], and XGBoost [38].

The NB classifier uses Bayes’ theorem to calculate the probability of input belonging to a class based on its features. It multiplies conditional probabilities to predict the class with the highest probability, showing reliable performance in genomics. SVM seeks a hyperplane to separate data into classes by maximizing the margin between it and the closest data points. This method accurately assigns new data points to their respective classes. LR models relationships between input variables and a binary outcome using a logistic function, providing probabilities between 0 and 1, which is valuable for genomics and binary classification. RF aggregates predictions from multiple decision trees, each trained on a data subset, ensuring robust classification. XGBoost iteratively adds trees to correct errors made by previous trees using gradient boosting, resulting in accurate predictions and reliability.

### MAC-ErrorReads evaluation stage

The machine learning models are evaluated using standard metrics such as accuracy, precision, recall, F1-score, MCC, and ROC. These metrics are defined in terms of True Positives (*TP*), False Positives (*FP*), False Negative (*FN*), and True Negative (*TN*). *TP* is the number of error-free reads that are correctly classified as error-free by the classification model. In other words, *TP* represents the instances where the model correctly identifies an error-free read as error-free. *FP* is the number of erroneous reads incorrectly classified as error-free by the classification model. In this case, the model mistakenly identifies a read containing errors as error-free. *FN* is the number of error-free reads incorrectly classified as erroneous by the classification model. Here, the model wrongly labels an error-free read as containing errors. *TN* is the number of erroneous reads correctly classified as erroneous by the classification model. In other words, *TN* represents the instances where the model correctly identifies a read containing errors as erroneous. Standard evaluation metrics for different machine learning models are defined in Table 2. To assess the efficacy of the trained machine learning models, we utilized reads obtained from real sequencing experiments that corresponded to the previously trained reference genomes. The primary objective was to classify each read as correct or incorrect using various machine learning models. However, a significant challenge arose from the fact that the reads in the sequencing experiments lacked explicit labels, and their accuracy levels were unknown beforehand. This uncertainty was attributed to the presence of diverse library preparation and sequencing biases that influenced the quality of the reads.

**Table 2:** Standard evaluation metrics for machine learning reads classification

Metric	Definition
*TP*	True Positives are error-free reads correctly classified as error-free
*FP*	False Positives are erroneous reads incorrectly classified as error-free
*FN*	False Negatives are error-free reads incorrectly classified as erroneous
*TN*	True Negatives are erroneous reads correctly classified as erroneous
*accuracy*	$\frac{{\left( {TP + TN} \right)}}{{\left( {TP + TN + FP + FN} \right)}}$
*precision*	$\frac{{\left( {TP} \right)}}{{\left( {TP + FP} \right)}}$
*recall*	$\frac{{\left( {TP} \right)}}{{\left( {TP + FN} \right)}}$
*F*1-*score*	$\frac{{2 \times r{\text{ecall}} \times p{\text{recision}}}}{{\left( {r{\text{ecall}} + p{\text{recision}}} \right)}}$
*MCC*	$\frac{TP \times TN - FP \times FN}{{\sqrt {\left( {TP + FP} \right) \times \left( {TP + FN} \right) \times \left( {TN + FP} \right) \times \left( {TN + FN} \right)} }}$

To address this issue, we aligned the reads to their reference genome by utilizing NGS aligners such as BWA [42] and Bowtie 2 [43]. The alignment statistics, including the total number of mapped reads and the alignment rate, are reported by Bowtie 2. The alignment score, computed for each read by BWA, allowed us to evaluate the accuracy of the reads based on the total number of bases aligned to the reference genome relative to the overall read length. Through a comprehensive analysis of the alignment scores, we were able to extract valuable insights regarding the accuracy level of the reads. Consequently, the corresponding labels of the reads were established by implementing specific threshold settings that varied based on the total number of mismatches, considering the total read length.

By comparing the predicted labels generated by MAC-ErrorReads with the true labels inferred from the alignment scores, we could effectively evaluate the performance of the trained models. This approach allowed us to assess how well the models classified the reads as correct or incorrect based on the alignment score-derived labels, providing crucial feedback on the models' effectiveness and reliability. Furthermore, the collection of correctly labeled reads could be subjected to additional analysis, where they are assembled using one of the NGS assemblers (i.e., Velvet [44]) that bypass the error correction stage. Subsequently, various assembly evaluation metrics [45] can be applied to assess the quality and accuracy of the assembled sequences. We extended our analysis by evaluating the assembly results with/without applying the error correction using a stand-alone tool (i.e., Lighter [13]). We then compared these assembly results with those filtered by the best reported machine learning model without error correction and with correcting the erroneously classified reads.

MAC-ErrorReads is implemented in Python 3.9, with the availability of libraries numpy, pandas, matplotlip, and sklearn. Its source code is freely available from the GitHub repository (https://github.com/amirasamy95/MAC-ErrorReads) under the MIT license.

### MAC-ErrorReads performance on *E. coli* dataset

The first genome used in the training process of the MAC-ErrorReads system is *Escherichia coli str. K-12 substr. MG1655 (E. coli)* with RefSeq accession NC_000913. Using *C* = 30X, = 4641652 bp, and *L* = 300 bp, the total number of paired-end reads used to cover the genome is $N \le 464165$. Machine learning models were trained using various *k*-mer sizes (7, 9, 11, 13, and 15) [46] on a dataset comprising 400,000 correctly reads labeled with 0 and 400,000 erroneous reads labeled with 1. To ensure that the labels assigned to the reads reflect its accuracy, we used a wgsim simulator to generate the required number of *N* = 800,000 paired-end reads with *L* = 300, and the error rate *e* = 0 for correct reads, and *e* = 1 for erroneous reads. We split the data into training (600,000 reads) and testing (200,000 reads). Subsequently, the training data is divided using a fivefold cross-validation scheme. The hyperparameters’ settings for different machine learning models are presented in Table 3.

**Table 3:** Hyperparameter settings for machine learning models

ML models	Hyperparameters
SVM	C = 1
RF	n_estimators = 100, min_samples_split = 2, min_samples_leaf = 1
LR	C = 1, penalty = L2, solver = lbfgs
NB	Alpha = 0.1
XGBoost	Max_depth = 3, n_estimators = 300, learning rate = 0.1

The performance of SVM, RF, LR, NB, and XGBoost on a simulated testing dataset (200,000 reads) is presented in Table 4, demonstrating the algorithmic performance across various *k*-mer sizes.

**Table 4:** Performance results of different machine learning models for the *E. coli* simulated dataset

Metrics	Accuracy					Precision					Recall					F1-score					MCC	ROC
*k*-mer size	7	9	11	13	15	7	9	11	13	15	7	9	11	13	15	7	9	11	13	15	11	11
SVM	1	1	1	1	1	1	1	1	1	1	1	1	1	1	1	1	1	1	1	1	0.99	0.99
RF	0.99	1	1	1	1	0.99	1	1	1	1	0.99	1	1	1	1	0.99	1	1	1	1	0.99	0.99
LR	0.99	1	1	×	×	0.99	1	1	×	×	0.99	1	1	×	×	0.99	1	1	×	×	0.99	0.99
NB	0.97	0.98	1	1	1	0.97	0.98	1	1	1	0.97	0.98	1	1	1	0.97	0.98	1	1	1	0.99	0.99
XGBoost	0.93	0.86	1	×	×	0.94	0.89	1	×	×	0.93	0.86	1	×	×	0.93	0.86	1	×	×	0.99	0.99

**Fig. 3:**
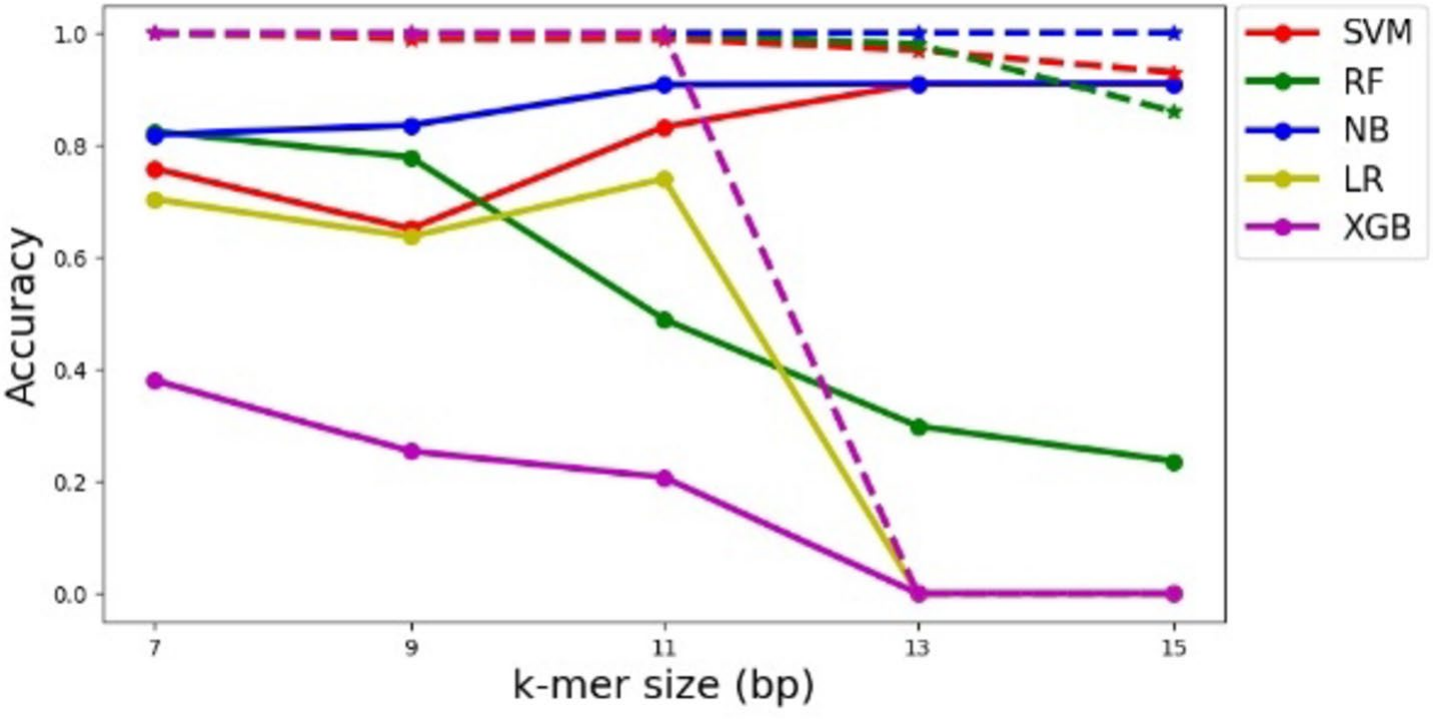
Accuracy results of different machine learning models for the *E. coli* simulated (dotted lines) and real (solid lines) datasets using different *k*-mer sizes

**Fig. 4:**
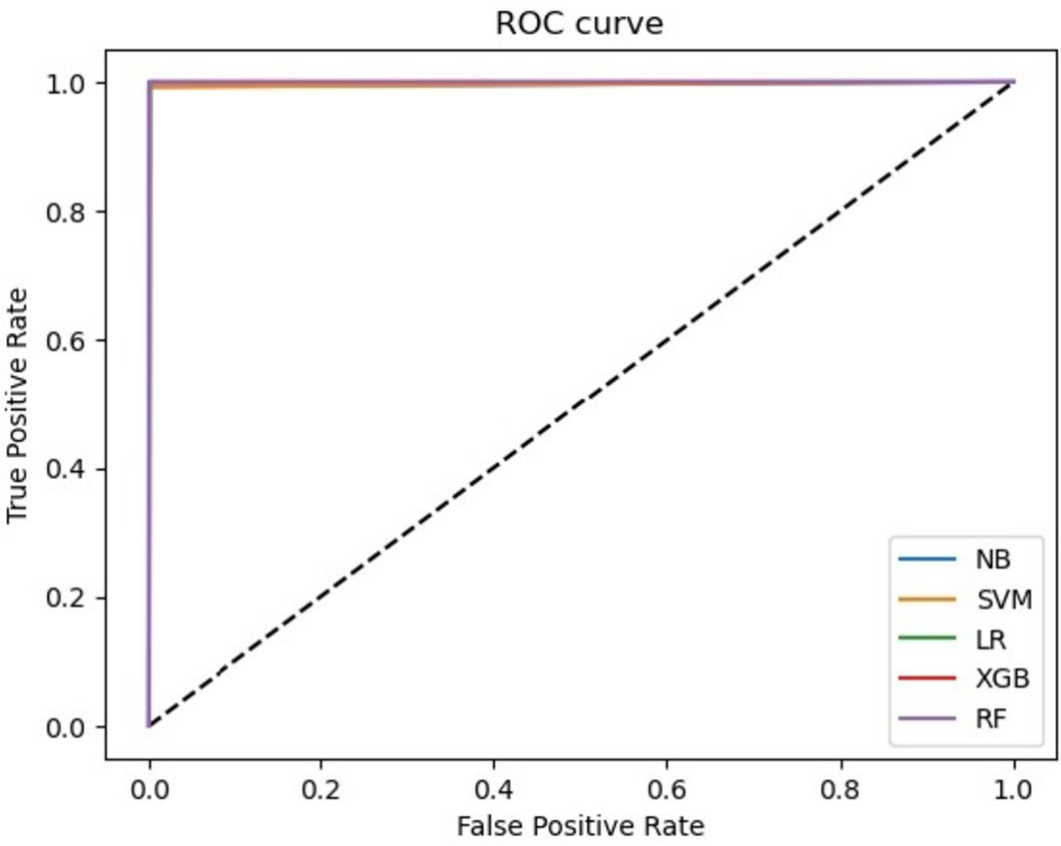
ROC curve of different machine learning models for the *E. coli* simulated dataset using *k* = 11

The accuracy, precision, recall, and F1-score are calculated using the equations provided in Table 2. Some reported results encountered issues, denoted by " × " indicating that these metrics could not be computed. Across all *k*-mer sizes, SVM consistently achieved a perfect score (1) for accuracy, precision, recall, and F1-score. Like SVM, RF and NB achieved very high scores for all metrics across most *k*-mer sizes, with a minor decrease observed when a small *k*-mer size (7 and 9) is used. LR and XGBoost addressed difficulties in handling high *k*-mer sizes (13 and 15), indicating the importance of selecting the appropriate* k*-mer size for the given dataset (see Fig. 3, represented by dotted lines). All models applied to an *E. coli* simulated dataset with a *k*-mer size of 11 achieved a value of 0.99 for both MCC and ROC, indicating a consistent and strong performance across the different machine learning models (see Fig. 4).

To evaluate the effectiveness of the *E. coli* trained models on reads from real sequencing experiments, we utilized a real dataset with accession number SRR625891. For the testing phase, we employed 100,000 reads from this sequencing experiment, where each read had a length of 90 bases. Tables 4, 5, 6, and 7 show the accuracy, precision, recall, and F1-score results of different algorithms for different *k*-mer sizes with different alignment thresholds.

**Table 5:** Performance of machine learning models on *E. coli* sequencing dataset (threshold: total aligned bases = 90 bp)

Metrics	Accuracy					Precision					Recall					F1-score				
*k*-mer size	7	9	11	13	15	7	9	11	13	15	7	9	11	13	15	7	9	11	13	15
SVM	0.76	0.65	0.83	0.91	0.91	0.81	0.68	0.89	1	1	0.91	0.92	0.92	0.91	0.91	0.86	0.78	0.91	0.95	0.95
RF	0.82	0.78	0.49	0.3	0.24	0.9	0.84	0.49	0.25	0.17	0.91	0.91	0.91	0.92	0.93	0.90	0.87	0.63	0.39	0.29
LR	0.70	0.64	0.74	×	×	0.75	0.66	0.78	×	×	0.91	0.92	0.92	×	×	0.82	0.77	0.85	×	×
NB	0.82	0.84	0.91	0.91	0.91	0.89	0.91	1	1	1	0.91	0.91	0.91	0.91	0.91	0.9	0.91	0.95	0.95	0.95
XGBoost	0.38	0.25	0.21	×	×	0.35	0.20	0.14	×	×	0.91	0.90	0.91	×	×	0.51	0.33	0.25	×	×

**Table 6:** Performance of machine learning models on *E. coli* sequencing dataset (threshold: total aligned bases = 80 bp)

Metrics	Accuracy					Precision					Recall					F1-score				
*k*-mer size	7	9	11	13	15	7	9	11	13	15	7	9	11	13	15	7	9	11	13	15
SVM	0.81	0.67	0.88	0.99	0.99	0.81	0.67	0.89	1	1	0.99	0.99	0.99	0.99	0.99	0.89	0.80	0.93	0.99	0.99
RF	0.88	0.83	0.49	0.26	0.18	0.9	0.84	0.48	0.25	0.17	0.99	0.99	0.99	0.99	0.99	0.94	0.91	0.65	0.4	0.29
LR	0.74	0.66	0.77	×	×	0.75	0.66	0.78	×	×	0.99	0.99	0.99	×	×	0.85	0.79	0.87	×	×
NB	0.88	0.9	0.98	0.99	0.99	0.89	0.91	1	1	1	0.99	0.99	0.99	0.99	0.99	0.93	0.94	0.99	0.99	0.99
XGBoost	0.36	0.21	0.15	×	×	0.36	0.20	0.14	×	×	0.99	0.99	0.99	×	×	0.52	0.34	0.25	×	×

**Table 7:** Performance of machine learning models on *E. coli* sequencing dataset (threshold: total aligned bases = 70 bp)

Metrics	Accuracy					Precision					Recall					F1-score				
*k*-mer size	7	9	11	13	15	7	9	11	13	15	7	9	11	13	15	7	9	11	13	15
SVM	0.81	0.67	0.88	1	1	0.81	0.67	0.88	1	1	0.81	1	1	1	1	0.89	0.80	0.94	1	1
RF	0.89	0.83	0.49	0.25	0.17	0.89	0.84	0.48	0.25	0.17	0.99	1	1	1	1	0.94	0.91	0.65	0.4	0.29
LR	0.74	0.66	0.77	×	×	0.74	0.66	0.77	×	×	1	1	1	×	×	0.85	0.79	0.87	×	×
NB	0.88	0.90	0.99	1	1	0.89	0.90	1	1	1	1	1	1	1	1	0.94	0.95	1	1	1
XGBoost	0.36	0.21	0.15	×	×	0.36	0.20	0.14	×	×	1	1	1	×	×	0.52	0.34	0.25	×	×

As the reads in real sequencing experiments lacked labels, we employed the BWA aligner to align the reads to the *E. coli* reference genome and extract their alignment scores from the resulting SAM files. Using the read length and the alignment score, which represents the total number of bases in the read aligned to the reference, we could establish thresholds to assign labels to the reads. This enabled us to compare the classification labels generated by various models with the actual labels computed by the aligner based on the alignment score. The results computed in Table 5, 6, 7, and 8 consider different thresholds for the total number of aligned bases in the read sequence to the reference genome (i.e., 90, 80, 70, and 60).

**Table 8:** Performance of machine learning models on *E. coli* sequencing dataset (threshold: total aligned bases = 60 bp)

Metrics	Accuracy					Precision					Recall					F1-score				
*k*-mer size	7	9	11	13	15	7	9	11	13	15	7	9	11	13	15	7	9	11	13	15
SVM	0.81	0.67	0.88	1	1	0.81	0.67	0.88	1	1	1	1	1	1	1	0.89	0.80	0.94	1	1
RF	0.89	0.83	0.48	0.25	0.17	0.89	0.84	0.48	0.25	0.17	1	1	1	1	1	0.94	0.91	0.65	0.4	0.29
LR	0.74	0.66	0.77	×	×	0.74	0.66	0.77	×	×	1	1	1	×	×	0.85	0.79	0.87	×	×
NB	0.88	0.90	0.99	1	1	0.88	0.90	1	1	1	1	1	1	1	1	0.94	0.95	1	1	1
XGBoost	0.36	0.20	0.14	×	×	0.36	0.20	0.14	×	×	1	1	1	×	×	0.52	0.34	0.25	×	×

The performance of machine learning models varied based on *k*-mer size and alignment thresholds. SVM demonstrated high accuracy (0.91–1) for *k*-mer sizes 13 and 15, effectively distinguishing true positive reads, but showed slightly lower accuracy for *k*-mer size 9 at lower thresholds. RF and LR generally achieved moderate accuracy, especially with smaller *k*-mer sizes, while LR faced challenges in computing accuracy for *k*-mer sizes 13 and 15. In contrast, NB consistently delivered exceptional performance across all *k*-mer sizes, with accuracy ranging from 0.8 to 1 for different alignment thresholds, highlighting its proficiency (see Fig. 3, represented by solid lines). Regarding precision, recall, and F1-score, SVM displayed consistent performance across *k*-mer sizes and thresholds, while RF and LR excelled with smaller *k*-mer sizes. NB consistently demonstrated high precision, recall, and F1-score across various configurations, indicating its accuracy in positive classifications. Conversely, XGBoost struggled to achieve high precision and F1-score levels across different settings, revealing limitations in classifying positive sequencing reads.

**Fig. 5:**
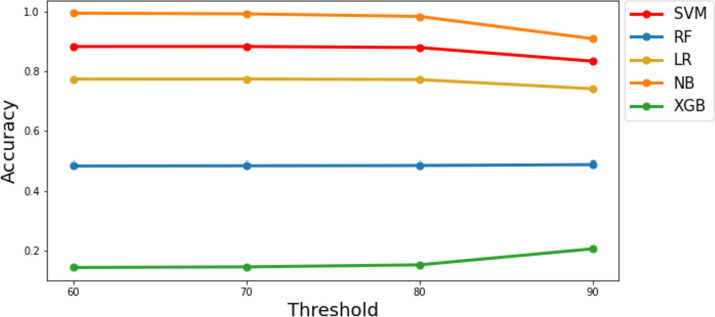
Accuracy results of different machine learning models for the *E. coli* real dataset using different thresholds for the aligned bases

Considering the previously recommended *k*-mer size of 11 for the *E. coli* dataset and the whole sequencing read length as a threshold for the total number of aligned bases (threshold = 90), NB yielded an accuracy value of 0.91, denoting that it correctly classified 91% of the sequencing reads in our dataset (see Fig. 5).

### MAC-ErrorReads performance on *S. aureus* dataset

**Fig. 6:**
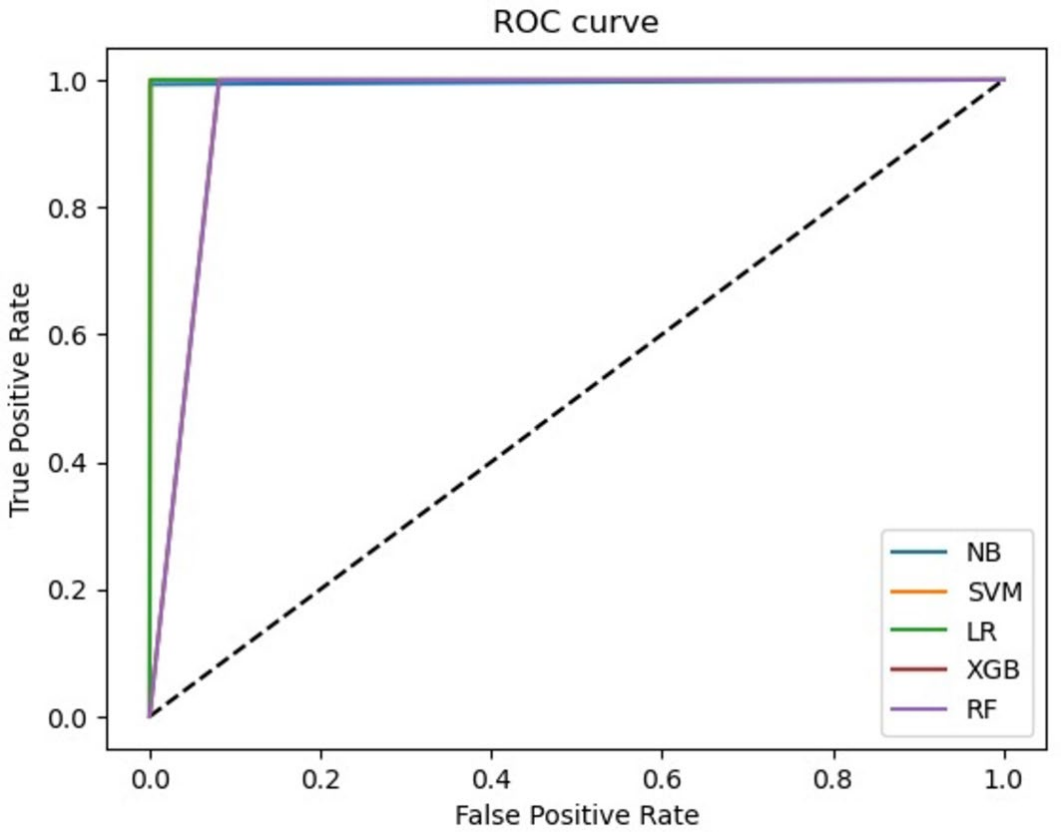
ROC curve of different machine learning models for the *S. aureus* dataset using *k* = 15

The second genome used in the training process is *Staphylococcus aureus (S. aureus)* from GAGE [47], the genome size is 2903081 bp and using *C* = 30X, and *L* = 101 bp, the total number of paired-end reads used to cover the genome is $N \le 862301$. We trained machine learning models using 400,000 correct reads labeled with 0 and 400,000 erroneous reads labeled with 1. We split the data into training (600,000 reads) and testing (200,000 reads), and then employ a fivefold cross-validation scheme to further partition the training data. We used *k*-mer size equals 15 to compute the *TF_IDF* feature values. Table 9 shows the performance results of different algorithms for the *S. aureus* testing dataset (200,000 reads). All machine learning models (SVM, RF, LR, NB, and XGBoost) show excellent performance on the *S. aureus* dataset with a *k*-mer size of 15, achieving high accuracy and consistent precision, recall, and F1-score values. SVM, LR, and NB stand out as they achieve perfect classification results, while RF and XGBoost are close behind, with only a slight deviation from perfect scores. Table 9 also presents MCC and ROC values for different machine learning models applied to the *S. aureus* dataset with a *k*-mer size of 15. Overall, SVM, LR, and NB models demonstrate consistent and strong performance in accurately classifying the *S. aureus* dataset (0.99 for both MCC and ROC), while RF (MCC = 0.92 and ROC = 0.96) and XGBoost (MCC = 0.92 and ROC = 0.95) exhibit slightly lower but still acceptable accuracy levels (see Fig. 6).

**Table 9:** Experimental results of different machine learning models for the *S. aureus* dataset (*k* = 15)

Metrics	Accuracy	Precision	Recall	F1-score	MCC	ROC
SVM	1	1	1	1	0.99	0.99
RF	0.96	0.96	0.96	0.96	0.92	0.96
LR	1	1	1	1	0.99	0.99
NB	1	1	1	1	0.99	0.99
XGBoost	0.95	0.95	0.95	0.95	0.92	0.95

We utilized a real dataset from GAGE for the same genome to evaluate the effectiveness of the *S. aureus* trained models on reads from real sequencing experiments. We employed 1,294,104 sequencing paired-end reads from this project, where each read had a length of 101 bases. For the correctly classified reads by different machine learning models, we conducted different analyses by assembling the reads using Velvet assembler that bypasses the error correction stage. The assembly results are evaluated by one of the assembly evaluation tools called QUAST [48]. The assembly evaluation metrics used to assess the quality of the assembled sequences, along with their values, are presented in Table 10. We ran QUAST using default settings with the minimum contig length for evaluating the assembly results at 500 bp. As stated in Table 11, the correctly classified reads by SVM and LR have the best assembly results for the total number of resulted contigs and N50 length. The NB and LR models correctly classified reads with the largest contig length (20,317 bp). NB has the best NG50 and genome coverage (94.2%) and the largest number of aligned bases to the reference genome (2,741,617 bp). All models have fairly similar counts of mismatches and indels in all aligned bases in the assembly results, with only slight variations.

**Table 10:** Assembly evaluation results of *S. aureus* dataset for different machine learning models

Dataset	NB correct classified reads of *S. aureus*	SVM correct classified reads of *S. aureus*	LR correct classified reads of *S. aureus*	RF correct classified reads of *S. aureus*	XGBoost correct classified reads of *S. aureus*
Metrics					
# contigs	2911	925	933	1425	1431
Largest contig	20,317	19,471	20,317	10,556	10,556
N50	2373	4228	4128	2269	2250
NG50	4329	3874	3852	1952	1924
Genome fraction (%)	94.206	92.415	92.483	85.761	85.475
#misassemblies	0	0	0	0	0
#misassembled contigs	0	0	0	0	0
Misassembled contigs length	0	0	0	0	0
# local misassemblies	2	3	3	4	5
#mismatches per 100 kbp	21.26	20.71	21.33	25.64	25.52
Total aligned length	2,741,617	2,688,962	2,691,135	2,495,928	2,487,833
#mismatches	583	557	574	640	635
#indels	10	12	9	9	11

**Table 11:** Comparison of assembly results with and without error correction of *S. aureus* reads, including the NB model performance

Dataset	*S. aureus* reads	*S. aureus* reads corrected by lighter	NB correct classified reads	NB correct classified reads + lighter for correcting erroneous classified reads
Metrics				
# contigs	3075	3086	2911	3084
Largest contig	22,555	22,555	20,317	22,555
N50	2952	2938	2373	2940
NG50	5832	5867	4329	5867
Genome fraction (%)	92.170	92.515	94.206	92.490
#misassemblies	1	1	0	1
#misassembled contigs	1	1	0	1
Misassembled contigs length	1826	1826	0	1826
# local misassemblies	3	3	2	3
#mismatches per 100 kbp	24.42	25.26	21.26	24.49
Total aligned length	2,682,193	2,691,757	2,741,617	2,690,983
#mismatches	655	680	583	659
#indels	17	19	10	19

We extended our analysis by evaluating the assembly results with/without applying the error correction in real sequencing experiments. We then compared these assembly results with those filtered by the NB machine learning model without error correction and with correcting the erroneously classified reads. This comparison allowed us to assess the accuracy of correctly classified reads by the NB model (see Table 11).

First, we assembled the entire dataset without error correction. Next, we employed a stand-alone error correction tool called Lighter [13] to correct the sequencing reads in the *S. aureus* dataset and subsequently conducted the assembly after the error correction stage. We then utilized Lighter to correct the erroneously classified reads by the NB machine learning model, combined them with the correctly classified ones by the same model, and performed assembly. Finally, we evaluated the assembly results produced by all these experiments with the results presented in Table 10 for the correctly classified reads by the NB model since it has the largest total aligned length and coverage corresponding to a *S. aureus* reference genome.

Table 11 provides a comprehensive comparison of assembly results for *S. aureus* reads under various scenarios, encompassing error correction using the stand-alone Lighter tool and classification by the NB machine learning model. Among these scenarios, the NB model's correctly classified reads yield the minimum number of assembled contigs, while the largest contig size, N50, and NG50 lengths are achieved by the reads that are corrected by Lighter, as well as those assembled without error correction, and the NB model's erroneously classified reads, which were subsequently corrected by Lighter and integrated with the correctly classified ones. Notably, the NB model’s correctly classified reads demonstrate the highest genome fraction percentage and the maximum total aligned length, signifying their extensive coverage of the *S. aureus* genome compared to results from other approaches. Moreover, the assembly results for the NB model’s correctly classified reads indicate the absence of misassemblies or misassembled contigs and exhibit a low rate of mismatches per 100 kbp. These favorable outcomes suggest that the NB model's classification significantly reduces assembly errors.

The correctly classified reads by the MAC-ErrorReads NB model are further analyzed and compared against various error correction tools, including Lighter, BFC, RECKONER, Fiona, Karect, Pollux, as well as the machine learning-based error correction tool CARE. It should be noted that these tools perform the error correction stage, while MAC-ErrorReads does not correct errors; instead, it filters out erroneous reads, retaining the correct ones for further downstream data analysis.

The correctly classified reads are benchmarked against those produced by different error correction tools, and alignment statistics are computed, indicating the total number of reads mapped by each tool and the percentage of reads successfully aligned to the reference genome (see Table 12). Tools like Lighter, BFC, RECKONER, and Fiona show similar alignment rates around 33–34%. Karect and Pollux exhibit higher alignment rates, with Pollux reaching around 37%, suggesting potentially better performance in read alignment. MAC-ErrorReads stands out with an alignment rate of 38.69%, significantly higher than most other tools, indicating strong performance in aligning reads. Bowtie 2 generated these statistics, accounting for reads aligned once or multiple times.

**Table 12:** Alignment statistics for various benchmarking error detection and correction tools

Tools	*S. aureus*		*H. Chr14*		*Arabidopsis thaliana Chr1*		*Metriaclima zebra*	
	Mapped reads*	Alignment rate (%)	Mapped reads*	Alignment rate (%)	Mapped reads*	Alignment rate (%)	Mapped reads*	Alignment rate (%)
Lighter	565,623	33.82	992,819	99.28	272,145	90.72	742,408	82.49
BFC	562,978	33.66	989,326	98.93	272,221	90.74	741,331	82.37
RECKONER	555,322	33.20	989,606	98.96	275,333	91.78	754,974	83.67
Fiona	548,248	32.78	957,850	95.78	265,828	88.61	722,217	80.59
Karect	588,395	35.18	991,623	99.16	275,393	91.80	753,414	83.71
Pollux	441,739	36.77	312,731	99.15	128,813	92.62	297,605	91.23
CARE**	541,616	32.38	990,305	99.03	272,150	90.72	744,147	82.68
MAC-ErrorReads***	540,246	38.69	959,644	99.14	262,880	90.87	699,018	83.76

*Bowtie 2 is utilized to generate these statistics, wherein the total number of mapped reads encompasses those aligned only once as well as those aligned multiple times

**CARE relies on RF machine learning model for error correction

***MAC-ErrorReads is a filtration tool that classifies reads as either correct or erroneous but does not perform error correction for the reads classified as erroneous

The correct and false classified reads by the NB model for the *S. aureus* genome undergo redundancy analysis to identify the duplicated reads within both sets. We utilized the Dedupe script from the BBMap tool [49], revealing 7033 duplicate reads, accounting for 1.48% of the classified reads across both positive and negative sets.

### MAC-ErrorReads performance on *Human Chromosome 14* dataset

The third genome used in the training process is *Human Chromosome 14 (H. Chr14)* from GAGE, the genome size is 88289540 bp, and using *C* = 30*X*, and *L* = 101 bp, the total number of paired-end reads used to cover the genome is $N \le 8828954$. We trained the NB machine learning model with *k* = 11 using 500,000 correct reads labeled with 0 and 500,000 erroneous reads labeled with 1. We split the data into training (700,000 reads) and testing (300,000 reads). Subsequently, the training data is divided using a fivefold cross-validation scheme. The NB model is chosen since it demonstrates consistent and strong performance in the classification and assembly results of the previous experiments. The NB model achieved the following performance metrics: accuracy (0.98), precision (0.98), recall (0.98), and F1-score (0.98). Additionally, reported values for the model include an MCC of 0.96 and an ROC of 0.98.

To evaluate the effectiveness of the *H. Chr14* trained model on real sequencing data, we utilized a real dataset from GAGE, targeting the same genome. Specifically, we employed 1 Mbp paired-end sequencing reads (*L* = 101 bp) from this project. The reads correctly classified by the MAC-ErrorReads model were then aligned to the *H. Chr14* reference genome using Bowtie 2, and alignment statistics were computed. Additionally, we conducted a benchmark comparison with various error correction tools mentioned previously (refer to Table 12). Notably, tools like Lighter, Karect, CARE, Pollux, and MAC-ErrorReads demonstrated alignment rates exceeding 99%. BFC and RECKONER achieved rates over 98%, while Fiona exhibited a slightly lower alignment rate of 95.78%.

The NB model's correct and false classified reads for the *H. Chr14* experience redundancy analysis, revealing 110 duplicate reads, accounting for 0.17% of the classified reads across both positive and negative sets.

### MAC-ErrorReads performance on *Arabidopsis thaliana* dataset

The MAC-ErrorReads is trained on the *Chr1 of Arabidopsis thaliana* genome with RefSeq accession NC_003070.9. The dataset size is 30,427,671 bp, and the total number of paired-end reads used to cover this genome is $N \le 3651321$, considering the *C* = 30*X*, *L* = 250 bp. We trained the NB machine learning model with *k* = 11using 200,000 correct reads labeled with 0 and 200,000 erroneous reads labeled with 1. The dataset set is split into 300,000 reads for training and 100,000 reads for testing. Subsequently, a fivefold cross-validation scheme is employed to further partition the training data. The NB model achieved an accuracy of 0.99, precision of 0.99, recall of 0.98, F1-score of 0.99, MCC of 0.98 and a ROC of 0.99. To assess the performance of the *Arabidopsis thaliana Chr1* trained model on real sequencing data from the same genome, we obtained 300,000 reads, each with a length of 250 bp from a sequencing run with accession number ERR2173372. The benchmarking results, comparing the performance with various error correction tools, are presented in Table 12.

Overall, Karect, RECKONER, and MAC-ErrorReads showed good alignment rates of 91.80%, 91.78%, and 90.87%, respectively, while also producing reasonable numbers of mapped reads to the reference genome. Pollux achieved a high alignment rate (92.62%) despite having fewer mapped reads compared to the other tools, as it excludes a substantial number of sequencing reads before initiating the error correction process.

The redundancy analysis of the correctly and falsely classified reads by the NB model for *Arabidopsis thaliana Chr1* revealed a total of 991 duplicated reads, accounting for 4.66% of the classified reads across both positive and negative sets.

### MAC-ErrorReads performance on *Metriaclima zebra* dataset

The MAC-ErrorReads is trained on *Metriaclima zebra* genome with RefSeq accession GCF_000238955.4. The dataset size is 957.5Mbp, and the total number of paired-end reads used to cover this genome is $N \le 284396638$, considering the *C* = 30*X*, *L* = 101 bp. We trained the NB machine learning model with *k* = 11 using 500,000 correct reads labeled with 0 and 500,000 erroneous reads labeled with 1. The dataset set is split into 700,000 reads for training and 300,000 reads for testing, followed by the employment of a fivefold cross-validation scheme to further partition the training data. The NB model achieved an accuracy of 0.96, precision of 0.97, recall of 0.96, F1-score of 0.96, MCC of 0.93 and a ROC of 0.96. To assess the performance of the *Metriaclima zebra* trained model on real sequencing data from the same genome, we obtained 900,000 reads, each with a length of 101 bp from a sequencing run with accession number SRR077289. The benchmarking results, comparing the performance with various error correction tools, are presented in Table 12.

Overall, MAC-ErrorReads, Karect, and RECKONER exhibited good alignment rates of 83.76%, 83.71% and 83.67%, respectively, while also generating reasonable numbers of mapped reads to the reference genome. Pollux achieved a high alignment rate (91.23%) despite having fewer mapped reads compared to the other tools, as it excludes a substantial number of sequencing reads before initiating the error correction process.

The redundancy analysis of the correctly and falsely classified reads by the NB model for *Metriaclima zebra* revealed a total of 29,392 duplicated reads, accounting for 22.82% of the classified reads across both positive and negative sets.

## Discussion

In this study, we introduced MAC-ErrorReads, a machine learning-assisted classifier designed to address the challenge of distinguishing erroneous from accurate reads in NGS datasets. MAC-ErrorReads converts the erroneous NGS read filtration process into a robust binary classification problem, where reads are classified as either ‘1’ for erroneous or ‘0’ for correct. Five supervised machine learning algorithms NB, SVM, RF, LR, and XGBoost were trained and tested using simulated and real data sets from the *E. coli*, GAGE *S. aureus*, *H. Chr14*, *Arabidopsis thaliana Chr1*, and *Metriaclima zebra* genomes. These algorithms were trained on a set of extracted features obtained through the computation of *TF_IDF* values for the set of identified *k*-mers from the sequencing data. The extracted features are computed without relying on expensive preprocessing stages such as MSA or multiple *k*-mers hashing techniques. We provided a theoretical limit on the number of reads used for a training process utilizing the Lander-Waterman sequencing coverage equation. Various evaluation metrics, including accuracy, precision, recall, F1-score, MCC, and ROC are computed based on alignment scores and used to assess the classification labels reported by the machine learning trained models.

For the simulated *E. coli* dataset, SVM consistently achieved a perfect score (1) for accuracy, precision, recall, and F1-score across different values of *k* for *TF_IDF* features computation. This indicates that SVM excelled in correctly classifying the sequencing reads, regardless of the *k*-mer size used. Similar to SVM, RF and NB achieved very high scores for all metrics across most *k*-mer sizes. A minor decrease in various evaluation metrics is observed when a small *k*-mer size (7 and 9) is used, indicating that the *TF_IDF* features with these specific *k*-mer sizes encounter difficulty discerning true positive instances. LR and XGBoost faced difficulties in handling high *k*-mer sizes (13 and 15), indicating the importance of selecting the appropriate *k*-mer size for the given dataset and the potential impact on different machine learning models' performance. All models applied to an *E. coli* simulated dataset with a *k*-mer size of 11 achieved a value of 0.99 for both MCC and ROC metrics. Overall, we recommend utilizing a *k*-mer size of 11 for the *E. coli* dataset, which is expected to yield satisfactory performance outcomes for all machine learning models.

We evaluated the effectiveness of the *E. coli* trained models on reads from real sequencing experiments considering different *k*-mer sizes and alignment thresholds. The accuracy results of SVM varies based on both k-mer size and the threshold for the number of aligned bases. Notably, it shows high accuracy (0.91–1) for *k*-mer sizes 13 and 15, indicating its effectiveness in distinguishing true positive sequencing reads. For other *k*-mer sizes, SVM achieves decent accuracy, though it drops slightly for *k*-mer size 9 at lower thresholds. RF and LR generally show moderate accuracy outcomes, primarily for smaller *k*-mer sizes. It is worth noting that LR experiences difficulty in computing accuracy for *k*-mer sizes 13 and 15. In contrast, NB delivers consistently exceptional performance across all *k*-mer sizes, with accuracy ranging from 0.8 to 1, considering various alignment thresholds. Conversely, XGBoost faces challenges in achieving satisfactory accuracy results when compared to other models. Additionally, it fails to produce accuracy outcomes for *k*-mer sizes 13 and 15.

In terms of precision, recall, and F1-score results, SVM exhibits consistent performance across various *k*-mer sizes and thresholds. On the other hand, RF and LR demonstrate favorable outcomes with smaller *k*-mer sizes as opposed to larger ones. Notably, LR experienced issues providing results for *k*-mer sizes 13 and 15. Conversely, NB demonstrates precision levels ranging from 0.88 to 1, as well as recall and F1-score levels ranging from 0.9 to 1 across diverse *k*-mer sizes and thresholds. This achievement highlights NB proficiency in accurate positive classifications, particularly evident through consistently high precision, recall, and F1-score across all configurations. However, XGBoost encounters difficulties in attaining high precision, and F1-score levels across various *k*-mer sizes and thresholds, featuring its limitations in effectively classifying positive sequencing reads.

Considering the previously recommended *k*-mer size of 11 for the *E. coli* dataset and the whole sequencing read length as a threshold for the total number of aligned bases (threshold = 90), NB yielded an accuracy value of 0.91, denoting that it correctly classified 91% of the sequencing reads in our dataset. This accuracy level highlights the NB proficiency in differentiating between correct and erroneous reads. NB attained a precision metric of 1, which signifies that when our model designated a sequencing read as erroneous, it was accurate 100% of the time. This is important in our context, as classifying accurate reads as erroneous could lead to data loss and erroneous conclusions. NB high precision score demonstrates its ability to maintain a low rate of false positives. The recall of the NB model was computed at 1, which signifies that our model effectively identified 100% of the actual erroneous sequencing reads. A high recall value in this context is vital, as it implies that NB model can capture a significant portion of erroneous reads, which is crucial for downstream data analysis. The F1-score of NB model, a balanced measure of precision and recall, was determined to be 0.95. This score signifies that NB model manages to strike a harmonious balance between precision and recall, indicating that it performs well in making accurate classifications while also identifying a substantial portion of erroneous reads.

For the *S. aureus* simulated dataset, all machine learning models (SVM, RF, LR, NB, and XGBoost) show excellent performance with a *k*-mer size of 15, achieving high accuracy and consistent precision, recall, F1-score, MCC, and ROC values. SVM, LR, and NB stand out as they achieve perfect classification results, while RF and XGBoost are close behind, with only a slight deviation from perfect scores. We tested the efficiency of the trained models by utilizing real sequencing reads from GAGE for the *S. aureus* genome and evaluated the assembly results of the correct classified reads by all models. The correct classified reads by SVM, and LR has the best assembly results for the minimum number of resulted contigs and the largest N50 length. The NB and LR models correctly classified reads with the largest contig length. NB has the best NG50 and genome coverage and the largest number of aligned bases to the reference genome. All models have fairly similar counts of mismatches and indels in all aligned bases in the assembly results, with only slight variations.

We also presented a comprehensive comparison of assembly results for *S. aureus* reads under different conditions, including error correction using the stand-alone Lighter tool and classification by the NB machine learning model. The minimum number of assembled contigs produced by the correctly classified reads by the NB model while the largest contig size, the largest N50 and NG50 lengths are produced by the reads corrected by Lighter as well as the reads assembled without error correction and the NB erroneously classified reads that were subsequently corrected by Lighter and combined with the correctly classified reads by the same model. The NB correct classified reads exhibit the highest genome fraction percentage and the largest total aligned length, indicating that they cover a larger portion of the *S. aureus* genome compared to the assembly results produced by the other approaches. Further, the assembly results of the NB correct classified reads show no misassemblies or misassembled contigs and have a low rate of mismatches per 100 kbp, which are positive outcomes, suggesting that the NB model's classification results help reducing the assembly errors. Generally, The NB correct classified reads demonstrate several advantages: they have a higher genome fraction percentage, lower mismatch rates, and a more extensive total aligned length compared to reads corrected by the stand-alone error correction tool Lighter and reads assembled without error correction. Additionally, they are free from misassemblies and misassembled contigs. These results suggest that the NB model's classification effectively identifies and retains the most accurate reads, improving assembly quality and genomic coverage for *S. aureus*.

The correctly classified reads from the MAC-ErrorReads NB model for the *S. aureus* dataset are compared against reads produced by error correction tools such as Lighter, BFC, RECKONER, Fiona, Karect, Pollux, and CARE. Unlike these tools that correct errors, MAC-ErrorReads filters out errors, preserving accurate reads for further analysis. Benchmarking these reads reveals that while Lighter, BFC, RECKONER, and Fiona show alignment rates of around 33–34%, Karect, and Pollux exhibit higher rates, especially Pollux at around 37%. Notably, MAC-ErrorReads stands out with an alignment rate of 38.69%, surpassing most tools. Additionally, redundancy analysis on correctly and falsely classified reads uncovers 7033 duplicate reads, constituting 1.48% of classified reads for the *S. aureus* genome.

For the *H. Chr14* simulated dataset, the NB model achieved an accuracy of 0.98, with precision, recall, and F1-score also at 0.98. Additionally, it obtained an MCC of 0.96 and a ROC of 0.98. Alignment statistics were computed for the correctly classified reads and compared with various error correction tools. Notably, Lighter, Karect, CARE, Pollux, and MAC-ErrorReads showed exceptional alignment rates of over 99%. BFC and RECKONER achieved rates above 98%, while Fiona had a slightly lower rate of 95.78%. Furthermore, a redundancy analysis of correctly and falsely classified reads by the NB model identified 110 duplicate reads, comprising 0.17% of the total classified reads across both positive and negative sets for *H. Chr14*.

For the *Arabidopsis thaliana Chr1* simulated dataset, The NB model achieved an accuracy of 0.98, precision of 0.99, recall of 0.96, F1-score of 0.98, MCC of 0.96 and a ROC of 0.98. The alignment statistics indicated that Pollux, Karect, RECKONER, and MAC-ErrorReads demonstrated good alignment rates of 92.62%, 91.80%, 91.78%, and 90.87%, respectively. For the *Metriaclima zebra* simulated dataset, The NB model achieved an accuracy of 0.99, precision of 0.99, recall of 0.98, F1-score of 0.99, MCC of 0.98 and a ROC of 0.99. The alignment statistics revealed that the Pollux error correction tool achieved a high alignment rate of 91.23%, despite having the lowest number of mapped reads. MAC-ErrorReads, Karect, and RECKONER demonstrated good alignment rates of 83.76%, 83.71%, and 83.67%, respectively, while also producing reasonable numbers of mapped reads to the reference genome. The redundancy analysis of correctly and falsely classified reads by the NB model revealed a total of 991 and 29,392 duplicated reads, accounting for 4.66% and 22.82% of the classified reads across both positive and negative sets for the *Arabidopsis thaliana Chr1* and *Metriaclima zebra* respectively.

The choice of *k*-mer size has a notable impact on the performance of the machine learning models, with some models (SVM, NB) showing better performance at larger *k*-mer sizes, while others (RF, LR, and XGBoost) may not be able to produce results at these larger sizes. Upon calculating the *TF_IDF*, a vector is generated, its length determined by the count of distinct *k*-mers. As the *k*-mer size increases, the number of unique *k*-mers grows, the vector's size expands correspondingly, demanding a substantial amount of RAM, which fails to compute the performance results for larger *k*-mer sizes for some models. The findings highlight the importance of selecting appropriate features (*k*-mers) for the dataset and the significance of using the right model for specific configurations.

## Conclusions

This study introduces MAC-ErrorReads, a machine learning-assisted classifier that effectively addresses the challenge of distinguishing erroneous from accurate reads in NGS datasets. Through extensive testing on both simulated and real datasets, we have demonstrated the remarkable performance of MAC-ErrorReads, particularly the NB model, which achieved high accuracy and precision in read classification. Applying MAC-ErrorReads to real sequencing data from *E. coli*, *S. aureus*, *H. Chr14*, *Arabidopsis thaliana Chr1* and *Metriaclima zebra* yielded good performance results, with the NB model consistently outperforming other algorithms. Furthermore, our mapping and assembly results analysis highlighted the benefits of correctly classified reads by the NB model, including comparable alignment rates, enhanced genome coverage, and reduced errors. By eliminating the need for expensive preprocessing stages and offering robust classification capabilities, MAC-ErrorReads streamlines NGS data analysis and enhances its quality. This research represents a significant step forward in the field of genomics, leveraging the power of artificial intelligence and machine learning to improve the accuracy and reliability of genomic analysis. The fusion of genomics and artificial intelligence, as exemplified by MAC-ErrorReads, contributes to the refinement of NGS data and opens up new possibilities for genetics, genomics, and personalized medicine research. As genomics continues to play a pivotal role in understanding genetic variations, gene expression patterns, and epigenetic modifications, tools like MAC-ErrorReads hold the potential to revolutionize the NGS data manipulation and interpretation. Future directions of this study include utilizing feature selection (Minimizers) and reduction stages, allowing the incorporation of other additional features, such as the distribution of quality scores. Also, the group of erroneously classified reads can be analyzed further by identifying potential contamination of genetic material by microorganisms such as bacteria, viruses, and fungal genomes during the sequencing experiments.

## Data Availability

Datasets used in this manuscript are the Escherichia coli str. K-12 substr. MG1655 (E. coli) [GenBank: NC_000913.3] and its corresponding real sequencing run [SRA: SRR625891]. The Staphylococcus aureus (S. aureus) and the Human Chromosome 14 (H. Chr14) from GAGE with their corresponding real sequencing reads are publicly available from the GAGE website (http://gage.cbcb.umd.edu/data/). The Arabidopsis thaliana chromosome 1 [GenBank: NC_003070.9] and its sequencing reads [SRA: ERR2173372]. The Metriaclima zebra [GenBank: GCF_000238955.4] and its sequencing reads [SRA: SRR077289]. MAC-ErrorReads publicly available from the GitHub repository: (https://github.com/amirasamy95/MAC-ErrorReads).

## Abbreviations

NGS: Next-generation sequencing
NB: Naive Bayes
SVM: Support vector machine
RF: Random forest
LR: Logistic regression
XGBoost: EXtreme gradient boosting
*TF_IDF*: Term frequency-inverse document frequency
PacBio: Pacific Biosciences
MSA: Multiple sequence alignment
ANNs: Artificial neural networks
RNN: Recurrent neural network
TF: Term frequency
IDF: Inverse document frequency
TP: True positives
FP: False positives
FN: False negative
TN: True negative
